# PREVALENCE OF POSTURAL CHANGES IN SCHOOL CHILDREN AND ADOLESCENTS

**DOI:** 10.1590/1413-785220233102e262255

**Published:** 2023-06-09

**Authors:** BRUNO BOARI DE RESENDE, PAULA SILVA ALMEIDA, MARCELO AUGUSTO SILVA, PATRÍCIA SAMARA SANTOS, MARCOS VINÍCIUS DE ÁVILA, ANDRÉA CARMEN GUIMARÃES, LAILA CRISTINA MOREIRA DAMÁZIO, PAULO CHAVES SALDANHA

**Affiliations:** 1. Center University Tancredo Almeida Neves-UNIPTAN, Physiotherapy Course, São João del Rei, Minas Gerais, Brazil.; 2. Federal University São João del-Rei – UFSJ, Department of Physical Education and Health Sciences -DCEFS, São João del Rei, Minas Gerais, Brazil.; 3. Federal University São João del-Rei – UFSJ and Center University President Tancredo Almeida Neves - UNIPTAN, São João del Rei, Minas Gerais, Brazil.; 4. Federal University Ouro Preto, Ouro Preto, Minas Gerais, Brazil.

**Keywords:** Posture, Body weight, Students, Body mass index, Adolescent, Scoliosis, Postura, Peso corporal, Estudantes, Índice de Massa Corporal, Adolescente, Escoliose

## Abstract

**Objective:**

Investigate the prevalence of postural changes and correlate them with body weight and the weight of schoolchildren’s backpacks in a school in the city of São João del-Rei-MG. Material and

**Methods:**

The study is an original type, with a cross-sectional design, where 109 schoolchildren of both sexes and mean age of 13 years were evaluated. The New York scale was used for posture analysis, measuring body weight, height, backpack weight, and Body Mass Index (BMI). The ANOVA statistical test and Pearson’s correlation test were used, considering a significance level of 0.05.

**Results:**

According to the results, the general average of the scores of postural problems was 68.7 points, with a predominance in the head, spine, hips, trunk, and abdomen. The regions of shoulder, feet, and neck presented mean scores below seven. The mean height was 1.61 m, body weight 56.03 kg, backpack weight 4.49 kg and BMI was 21.51 kg/m.

**Conclusion:**

Postural alterations are highly prevalent among the evaluated students. The most affected body segments are the head, spine, hips, trunk, and abdomen. However, this finding was not related to the weight of the backpacks or the students’ body weight. However, different parameters must be used to analyze the factors that may be related to such findings, such as ergonomic changes, inadequate habits, growth spurt, among others. Evidence Level III,Cross-sectional Observational Study.

## RESUMO

**Objetivo:**

Investigar a prevalência de alterações posturais e correlacionar com o peso corporal e o peso das mochilas dos escolares em uma escola no município de São João del-Rei-MG.

**Métodos:**

O estudo é do tipo original, com delineamento transversal onde foram avaliados 109 escolares, com média de idade de 13 anos, de ambos os sexos. A escala de Nova Iorque foi utilizada para análise de postura, medição do peso corporal, altura, peso da mochila e Índice de Massa Corporal (IMC). Foi utilizado o teste estatístico ANOVA e o teste de correlação de Pearson, considerando o nível de significância de 0,05.

**Resultados:**

De acordo com os resultados, a média geral dos escores dos problemas posturais foi de 68,7 pontos, com predomínio na região da cabeça, coluna, nos quadris, no tronco, e no abdômen. As regiões dos ombros, pés e pescoço apresentaram médias de escores menores que 7. A média da altura foi de 1,61m, do peso corporal de 56,03kg, do peso das mochilas de 4,49 kg e 21,51 kg/m do IMC.

**Conclusão:**

Conclui-se que existe uma alta prevalência de alterações posturais entre os escolares avaliados. Sendo que, os segmentos corporais mais comprometidos são, a cabeça, a coluna vertebral, os quadris, o tronco e o abdômen. No entanto, esse achado não foi relacionado ao peso das mochilas ou ao peso corporal dos escolares. Assim, diferentes parâmetros devem ser utilizados para analisar os fatores que podem estar relacionados a tais achados, como alterações ergonômicas, hábitos inadequados, estirão de crescimento, entre outros. Nível de Evidência III, Estudo Transversal Observacional.

## INTODUCTION

Postural deviations are prevalent problems in the adult population but are also identified in children and adolescents. The most common postural alterations in the population are anteroposterior changes (scoliotic manifestations), dorsal hyperkyphosis, and lumbar hyperlordosis.^1^

These postural deviations may be related to muscle imbalances that cause diversions at the positional or structural levels, *e.g* ., if the individual remains for a long time in a specific position, such as sitting, lying down, or standing.^2^ Among other factors, the ones that stand out most when associated with postural changes and lower back pain in schoolchildren are sex,^1 , 3 , 4^ body composition, excessive loads imposed on the individual daily, and the time spent in a specific position.^2^

Recent studies have found associations between the way students carry school bags and their total weight - below or above 10% of their total body weight^4 , 5^ and lower back pain.^3^ In the study by Barbosa et al.^6^ associations were identified between backpack weight and postural changes among 5th and 9th grade students. However, the influence on postural changes in children and adolescents has not yet been elucidated.

The present study is extremely important because it investigated the prevalence of postural changes among schoolchildren and adolescents in São João del-Rei-MG, Brazil, seeing that such alterations develop in this age group, causing significant musculoskeletal limitations in the adult phase of these individuals. The identification and prevention of these disorders can benefit these children and adolescents regarding the awareness of how backpacks should be transported and how important an adequate body composition is for the quality of life of students.

Based on this knowledge, it will be possible to guide public health programs that can intervene early to improve the quality of life of these children and adolescents.

Thus, the present study aimed to investigate the prevalence of postural changes and analyze whether such changes correlate with the body weight or backpack weight of schoolchildren and adolescents aged between 11 and 15 years old in a school in the city of São João del-Rei-MG, Brazil.

## METHODS

This descriptive, cross-sectional study was carried out in August 2019 in a public school in the city of São João del-Rei, Minas Gerais, Brazil.

### Sample

The evaluated school has 150 students, and 109 students were evaluated in August 2019 (punctual prevalence), aged between 11 and 15 years (average age 13 years) and of both sexes, who agreed to participate in the present study. Their guardians signed the Free and Informed Consent Term at the time of registration, according to Resolution No. 466/2012 of the National Health Council. The anonymity and confidentiality of the research subjects’ data were guaranteed.

### Instruments and Procedures

The New York scale was used for the postural assessment of the students, where 10 different body segments were analyzed, adopting the following scores: 10 for normal posture; 5 for moderate postural alterations, and 0 for severe postural alterations. The scale is visual where the observer identifies the body alignment of the individual who is positioned in the anterior, profile and posterior view. It is a scale widely used among researchers in the area. The postural classification was obtained by adding the scores of each item. Scores between 56-65 were considered normal posture; between 40-55 moderate postural change, and between 01-39 points severe postural change. Each individual’s posture was evaluated in the dorsal and anterior view (frontal plane), including the head, shoulders, spine, hips, feet, and plantar arch regions, and in the lateral view (sagittal plane), including the neck, chest, shoulders, thoracic spine, torso and pelvis, lumbosacral spine, and abdomen. The postural assessment lasted an average of 2 minutes. First, a screen was placed, and a 4-meter fixed support plumb was hung in front of the screen. A line of adhesive tape was placed on the floor directly below the plumb line, perpendicular to the screen. A distance of three meters from the examiner was considered. Each examined individual was positioned between the screen and the plumb line, facing the screen for the first part of the evaluation and then in profile position for the second part of the evaluation. The plumb line passed directly through the midline of the students’ chest.^7^

Another trained evaluator measured their height and body weight. In order to assess height, a portable stadiometer for adults was used, with a retractable tape measure with an extension of up to 210 cm. A portable electronic scale was used to determine the students’ body weight, with a capacity of 150 kilograms (kg) and a graduation of 100 grams (g). At the same time, another evaluator weighed the students’ backpacks containing school supplies using a portable electronic scale, with a capacity of 150 kg and a graduation of 100 g.

### Statistical analysis

The data were assessed by analysis of variance (ANOVA) and Pearson’s correlation test, considering a significance level of 5%. The analysis was performed using the GraphPad Prism 5.0 statistical program.

## RESULTS

The data of the present study identified that all the children evaluated presented some alteration or postural deviation in some region of the body. The overall mean score of postural changes found was 68.7 points. Significant changes were observed in the head region, with a score of 7.5; in the spine, with a score of 7.45; in the hips, with 7.36; in the torso, with 7.77; and in the abdomen, with 7.86. The shoulders, feet, and neck regions had mean scores lower than 7, as shown in Table 1 .

**Table 1:** Mean scores of the postural evaluation scale per body segment.

Analyzed Body Regions	Mean Scores of the Scale
	
Head	7.5
Shoulders	5.86
Spine	7.45
Hips	7.36
Feet	5
Neck	6.54
Chest	6.68
Torso	7.77
Abdomen	7.86
Lumbar	6.64

**Figure 1:**
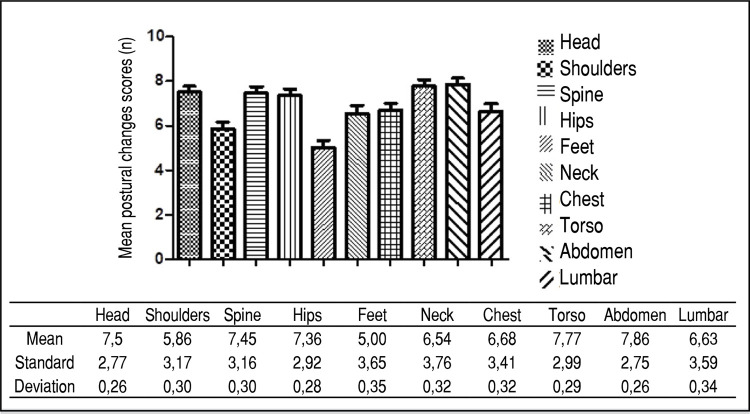
Mean postural changes of the body segments.

The data presented in Figure 1 demonstrate a significant difference between the mean scores of the postural changes of the analyzed body segments (p=0.00018; F=8.7431).

General data related to height, body weight, and the weight of students’ backpacks were analyzed, as shown in Table 2 , where the following values were observed: mean height of 1.61 m; mean body weight of 56.03 kg; mean backpack weight of 4.49 kg. The mean BMI was 21.51 kg/m.

**Table 2:** Means of the general parameters analyzed among the students.

Height (m)	1.61
Body Weight (kg)	56.03
Weight of the Backpacks (kg)	4.49
Total Score on the Postural Changes Scale (No.)	68.67
BMI (kg/m)	21.51

There was no correlation between the postural changes x backpack weight (p=0.2765; r=0.10516); body weight x backpack weight (p=0.8690; r=-0.015976); body weight x postural changes (p=0.7902; r=0.025651). There was, however, a correlation between body weight x height (p=0.00013; r=0.47214). A difference was observed among the means of the postural changes analyzed (p=0.0028; F=8.7431).

## DISCUSSION

The data obtained in the present study showed that the overall mean of the postural changes scale did not present high means among the children, in which the final score was 68.67 points. However, significant changes were observed in regions such as the head, spine, hips, torso, and abdomen. This corroborates the study by Ugras *et al* . (2010),^3^ where postural changes were also evidenced among children between 10 and 14 years old. In that study, 72.2% of the children had scoliosis (Cobb) angles between 10 and 20º, while 27.3% had Cobb angles greater than 20º. The study by Chaves *et al* . (2017)^2^ also revealed a high incidence of postural changes in the cervical region, the head, shoulders, thoracic spine, the lumbar area, and the lower limbs among students between 10 and 18 years old.

According to Reis-Diniz *et al* . (2012),^8^ during adolescence, the growth spurt occurs, and ergonomic, genetic, and lifestyle-related factors can favor the appearance of postural changes at this stage of life.

Another event that favors the occurrence of postural changes is obesity. According to Brandalize and Leite (2010),^9^ obesity worsened the posture of adolescents, contributing to the appearance of postural changes. In the study by Barbosa et al. (2019)^6^ higher weights were found in the backpacks of overweight and obese students.

In the present study, obesity was not evidenced among the children analyzed, where the mean body weight was 56 kg and the BMI was 21.51 kg/m for children with a mean age of 13 years. No correlation regarding postural changes, body weight, and the BMI of the children evaluated was found since the correlation index was 0.0089 (p>0.05).

According to the Brazilian Society of Pediatrics (2017), children with BMIs of more than 21.5 and a mean age of 13 years are considered overweight (Ministry of Health, 2017).^10^ Bravin (2016)^11^ stated in her study that the prevention of childhood obesity with physical activity is essential to reduce diseases in this population. The author mentioned that the appearance of diseases such as metabolic disorders, diabetes, and postural changes is common in this population.

In the present study, no correlation was found between backpack weight and the children’s postural assessment scores. In the studies by Mattioli et al. (2017)^12^ and Batista et al. (2016),^4^ the authors stated that the ideal weight for backpacks should be 10% of the student’s body weight. In the two studies, it was observed that most of the evaluated students carried backpacks weighing more than 10% of their body weight.

The mean weight of the student’s backpacks herein was 4.5 kg, which corresponds to 8% of the students’ body weight. Thus, such percentage does not significantly impact their posture, indicating that the postural changes evidenced in this study were not related to the mean weight carried in the students’ backpacks. In the study by Alami et al (2020),^13^ weights were found in students’ backpacks that were greater than 10% of their body weight. In the study by Kafle (2020),^14^ weight overload in students’ backpacks was also evidenced, correlated with the presence of musculoskeletal pain. The author concluded that there a correlation between excessive weight carried in the backpack with the appearance of postural changes and pain among students.

In the study by Lekpa et al. (2021)^15^ who evaluated 1070 schoolchildren in Douala, Cameroon, no correlations were observed between the weight of backpacks and the presence of low back pain. In study, the mean weight of backpacks below 10% of the students’ body weight was also found, and the factors associated with low back pain were female sex, practice of competitive sports, sitting position and low back pain in the evaluated family members.

In the work by Alfageme-García et al. (2020)^16^ carried out among students who had a neutral foot and carried high weights in their backpacks, it was evidenced the development of pronated foot among students after 3 years.

Postural changes in students may have different triggering factors, and body weight and backpack weight are not parameters directly related to the appearance of these changes.^17 - 20^

### Study limitations

One limitation in the present study was that it was carried out at only one school in the interior of Minas Gerais, Brazil. Further studies are needed in other elementary and secondary education institutions, as well as in private institutions, for comparisons with the data obtained at this public institution.

## CONCLUSION

It is concluded that there is a high prevalence of postural alterations among the evaluated students. The most affected body segments are the head, spine, hips, trunk and abdomen. However, this finding was not related to the weight of the backpacks or the body weight of the students. Thus, different parameters must be used to analyze the factors that may be related to such findings, such as ergonomic changes, inappropriate habits, growth spurt, among others.
